# Estimating red fox density using non-invasive genetic sampling and spatial capture–recapture modelling

**DOI:** 10.1007/s00442-021-05087-3

**Published:** 2021-12-02

**Authors:** Lars K. Lindsø, Pierre Dupont, Lars Rød-Eriksen, Ida Pernille Øystese Andersskog, Kristine Roaldsnes Ulvund, Øystein Flagstad, Richard Bischof, Nina E. Eide

**Affiliations:** 1 Norwegian Institute for Nature Research, Høgskoleringen 9, 7034 Trondheim, Norway; 2 Faculty of Environmental Sciences and Natural Resource Management, Norwegian University of Life Sciences, Universitetstunet 3, 1430 Ås, Norway; 3 Present Address: Centre for Ecological and Evolutionary Synthesis (CEES), The Department of Biosciences, University of Oslo, Blindernveien 31, 0371 Oslo, Norway

**Keywords:** Red fox, Density, Spatial capture–recapture, Non-invasive genetic sampling

## Abstract

Spatial capture–recapture modelling (SCR) is a powerful tool for estimating density, population size, and space use of elusive animals. Here, we applied SCR modelling to non-invasive genetic sampling (NGS) data to estimate red fox (*Vulpes vulpes)* densities in two areas of boreal forest in central (2016–2018) and southern Norway (2017–2018). Estimated densities were overall lower in the central study area (mean = 0.04 foxes per km^2^ in 2016, 0.10 in 2017, and 0.06 in 2018) compared to the southern study area (0.16 in 2017 and 0.09 in 2018). We found a positive effect of forest cover on density in the central, but not the southern study area. The absence of an effect in the southern area may reflect a paucity of evidence caused by low variation in forest cover. Estimated mean home-range size in the central study area was 45 km^2^ [95%CI 34–60] for females and 88 km^2^ [69–113] for males. Mean home-range sizes were smaller in the southern study area (26 km^2^ [16–42] for females and 56 km^2^ [35–91] for males). In both study areas, detection probability was session-dependent and affected by sampling effort. This study highlights how SCR modelling in combination with NGS can be used to efficiently monitor red fox populations, and simultaneously incorporate ecological factors and estimate their effects on population density and space use.

**Supplementary Information:**

The online version contains supplementary material available at 10.1007/s00442-021-05087-3.

## Introduction

Reliable information on animal population status, including population size and density, is crucial for wildlife research and management (Kämmerle et al. 2018). However, estimating population size and density is challenging. This is especially true for predators, because they often occur at low densities, are elusive, and inhabit areas that may also be difficult to survey due to inaccessibility or rough terrain (Kery et al. 2011). Predators are also often of management concern due to their conservation status or conflict potential with humans through direct threat, depredation of livestock, competition for game species (Estes 1996), or spreading pathogens (Moore et al. 2010b).

The red fox (*Vulpes vulpes*) is a highly adaptable and opportunistic mesopredator with a broad ecological niche and variable diet, including both wild and domestic vertebrates (Dell'Arte et al. 2007; Killengreen et al. 2011). It is the most widely distributed carnivore in the world and is commonly found in a wide array of habitats. It is also considered invasive or overabundant across much of its geographic range (Larivière and Pasitschniak‐Arts 1996). The species’ ongoing geographic expansion is of management concern due to deleterious effects on populations of other species. This includes intraguild competition with arctic fox (*Vulpes lagopus*; Frafjord et al. 1989), and predation on threatened species like the lesser white-fronted goose (*Anser erythropus*; Aarvak et al. 2017), and game species like forest birds (Doherty et al. 2016; Jahren 2017; Skrede 2016; Smedshaug et al. 1999). The red fox is also a vector of zoonotic pathogens that can pose risks for domestic animals and humans (Hodžić et al. 2016; Víchová et al. 2018; Laurimaa et al. 2016). Despite the importance of the red fox for wildlife management, a few practical methods are available for estimating population size and densities, required to evaluate effects of management actions (Wegge et al. 2019). Because direct observation of the red fox is difficult (Vine et al. 2009), methods used to monitor red fox populations have mainly been based on indirect measures, including culling indices (Smedshaug et al. 1999), snow tracking (Wegge and Rolstad 2011), fecal counts (Cavallini 1994; Webbon et al. 2004), mapping of active dens (Lindström 1989; Lindström et al. 1994), and camera trap visits (Hamel et al. 2013; Henden et al. 2014). These methods assume that the measured indices are directly proportional to the population parameter of interest, be it population size or density. This relationship is, however, often unknown, and thus, the reliability of these methods is difficult to evaluate (O'Connell et al. 2010; Sollmann et al. 2013).

An alternative approach is capture–recapture (CR) methodology. CR methods are widely used for estimating animal population parameters (Silvy 2012). CR uses multiple captures of the same individual, identified by natural or artificial means, to make extended inferences at the population level. An important advantage of these methods is their ability to account for imperfect and variable detection probability (Amstrup et al. 2010; Royle and Young 2008). Conventional CR, however, exhibits difficulty associated with estimating population density due to movements of animals into and out of the study area, which often leads to erroneous inferences (Royle and Young 2008; Royle et al. 2018).

Unlike conventional CR, spatial capture–recapture (SCR) incorporates a spatially explicit component in the model that accounts for spatial heterogeneity in detection probability of individuals. SCR can therefore estimate density as an explicit parameter (Royle et al. 2013). In addition, SCR models allow for the incorporation of ecological factors, such as sex or habitat characteristics, and estimate effects of these on population density and animal space use. SCR is also well suited for use in combination with non-invasive sampling methods, such as camera trapping and non-invasive genetic sampling (NGS) data (Mumma et al. 2015; Royle et al. 2013). NGS in combination with SCR methods has recently become a popular tool to monitor wide-ranging carnivores at large scales (Bischof et al. 2020). Recent studies also support use of these methods to monitor mesopredators when applied at appropriate spatial scales (Morin et al. 2016; Wegge et al. 2019).

The goal of the present study is to assess the combination of non-invasive genetic sampling with spatial capture–recapture for estimating red fox density, and explore the role of individual and spatial variables on density, space use, and detectability. We use data from two different study areas in Norway with different habitat and climate characteristics.

## Materials and methods

### Study areas

**Fig. 1:**
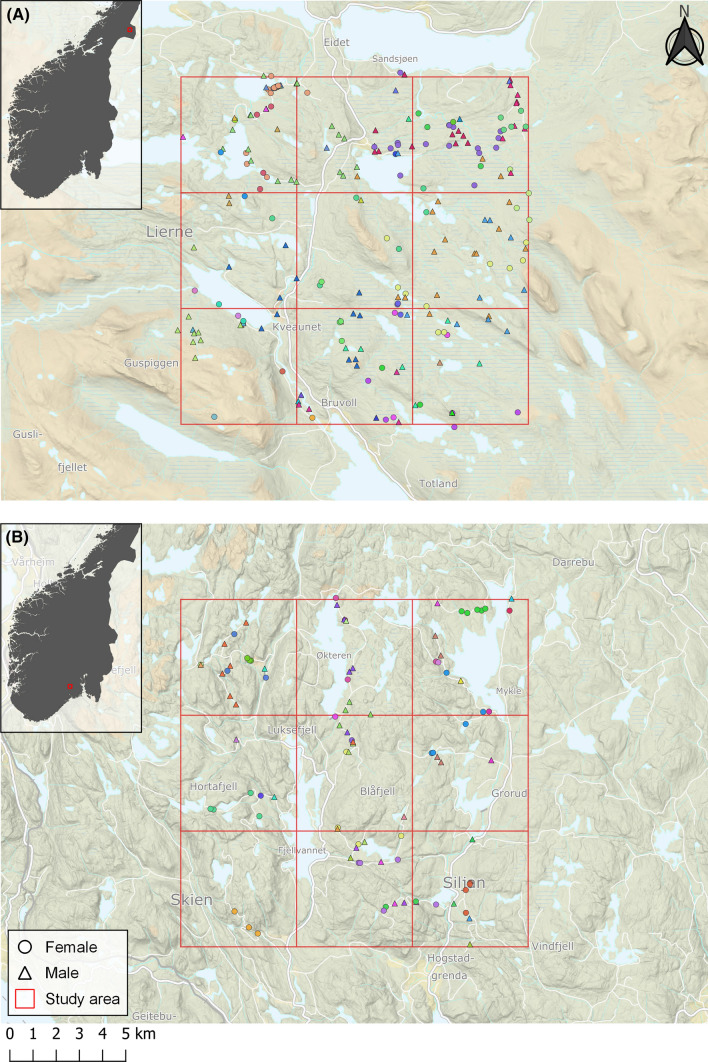
Map of the two 225 km^2^ study areas in **A** Lierne in central Norway and **B** Skrim in southern Norway. The study areas are shown with a 5 × 5 km grid with locations of all fecal, urine, and hair samples included in genetic analysis to identify individual red foxes, and subsequently used for estimating red fox density. Samples of the same colour represent samples from the same individual. Inset panels show each study area’s location in Norway

The first study area (“Lierne”) was established in Lierne, Trøndelag in central Norway (64.353° N, 13.659° E; Fig. 1A), where a pilot study was conducted in 2016. It consists of an undulating terrain between 500 and 950 m a.s.l. with mixed forests and protruding unforested crests, and a mean forest cover of 50%. Norway spruce (*Picea abies*) dominates the forests with interspersed Birch (*Betula spp*.) and Scots pine (*Pinus sylvestris*) (Moen 1998). Parts of the study area are subjected to commercial clear-cut forestry, and small settlements are scattered along the main road going through the study area. Parts of the region are used by semi-domestic reindeer (*Rangiferus tarandus*) for perennial pastures in addition to moose (*Alces alces*) and roe deer (*Capreolus capreolus*), and a diverse carnivore community, including arctic fox (*Vulpes lagopus*), wolverine (*Gulo gulo*), brown bear (*Ursus actos*), lynx (*Lynx lynx*), and pine marten (*Martes martes*; Gomo et al. 2017, 2020).

The second study area (“Skrim”) was established in 2017 near Skrim, Viken in southern Norway (59.391° N, 9.590° E; Fig. 1B). This study area is located between 400 and 675 m a.s.l., and is comparable to Lierne in terms of species composition (Østbye et al. 1989) and forestry practice (Moen 1998), but with denser forest cover (85%), rougher topography, and no unforested crests. The fauna in Skrim is less documented but comparable to Lierne, though the study area is located outside the range of wolverine, arctic fox, and reindeer. Human occupancy along the main roads is similar in both study areas (Norwegian Mapping Authority 2020), but the human population in adjacent settlements is substantially higher in Skrim municipality which includes a city (Skien) of 55 000 inhabitants. By contrast, the population of the entire municipality of Lierne is 1355 inhabitants (Statistics Norway 2020). Both study areas are 15 × 15 km (225 km^2^; Fig. 1).

### Data collection

Scats, urine, and hair from red fox were collected during February and March in 2016, 2017 and 2018 in Lierne, and in 2017 and 2018 in Skrim. Both study areas were divided into 5 × 5 km grids to guide the allocation of search effort. Sampling was predominantly done by the same local hunters each year and primarily focused along snow covered dirt roads, snowmobile tracks, and skiing tracks. Urine samples were collected by placing spruce sticks (40–60 cm in length) for foxes to urinate on at an interval of approximately 500 m along sampled roads and tracks. Each transect was sampled at least twice each year. Scat, urine, and hair samples were handled with gloves and plastic cutlery to avoid contamination of DNA, and placed in plastic vials containing silica gel or urine preservative fluid and paper envelopes, respectively, for preservation of DNA and storage for later analysis. All samples were dated and corresponding UTM coordinates were recorded with a handheld GPS unit.

### DNA extraction, amplification, and genotyping

The genetic analyses were undertaken at the Norwegian Institute for Nature Research (NINA) in Trondheim, Norway. DNA was extracted from 314 scat, 448 urine, and 23 hair samples (Table S1) using the FastDNA™ Spin Kit for Soil, the Norgen Biotek Urine DNA Isolation Kit (Slurry Format) and the Maxwell® 16 Tissue DNA Purification Kit, respectively, following the manufacturer’s protocols. To confirm red fox samples, two PCR runs followed by capillary electrophoresis were performed for each sample using the species identification method described by Dalén et al. (2004). Samples from other species than red fox were excluded from further analysis. All confirmed red fox samples were genotyped with 14 microsatellite markers, including a marker for sex determination (Moore et al. 2010a). To account for genotyping errors in low-quality samples (Fig. S1 and S2), three replicates per sample and marker were applied.

Consensus genotypes were assigned to each sample based on consistency across all three replicates for homozygote markers and at least two for heterozygotes. This procedure minimizes the risk of genotyping errors caused by allelic dropout and false alleles (Taberlet et al. 1996). To identify reliable genotypes, we assigned each sample a quality index (QI), calculated as the proportion of consistent gene scores across all three replicates (Miquel et al. 2006). Samples with a mean QI of 0.70 or above were retained for subsequent individual identification. Finally, we assigned identities using Allelematch, an R package for identifying unique multilocus genotypes where genotyping error and missing data may be present (Galpern et al. 2012), in R version 3.6.0 (R core team 2019).

### Spatial capture–recapture

#### General description

We estimated red fox densities for each study area using spatial capture–recapture (SCR) models. SCR models are hierarchical models composed of a submodel for the distribution of individuals in space, i.e., density (D), and a submodel for the detection of these same individuals, conditional on their location. SCR models assume that animals move around a central point referred to as the activity centre (AC). Density is modelled as the distribution of ACs over an area referred to as the state space that encompasses the surveyed area surrounded by a buffer large enough to include the AC location of any individual that could have been exposed to sampling (Royle et al. 2013). Density may be modelled as a function of spatially explicit covariates (Borchers and Efford 2008). SCR models usually assume that the detection probability of an individual declines with distance to an individual's AC. The most common detection model is the half-normal function, which has two parameters. The scale parameter (σ) describes how fast the detection probability decreases with distance, and the baseline detection probability (p0) describes the probability to detect an individual at the exact location of its AC. Both the scale parameter and the baseline detection probability can be related to different individual or spatial covariates to account for potential heterogeneity in detection (Royle et al. 2013). The detection model also implies a model of space use that is closely linked with home-range size through 1) movement of an individual about its home-range and 2) detection being proportional to space use in the vicinity of a detector. We can thus use SCR models to derive sex-specific home-range size estimates (i.e., the circular area encompassed by the 95% vertex of the utilization distribution) directly from the scale parameter σ using the Chi-square distribution with two degrees of freedom (Royle et al. 2013).

#### State space, detectors, and SCR data

Models were run separately for each study area, and therefore, the state space and potential detection locations, i.e., detectors, were also study area-specific. Detectors were defined as the centres of 500 × 500 m grid cells covering each 225 km^2^ study area (*N* = 900; Fig. S3 and S4). The state space for each study area was defined as a grid of 500 × 500 m cells covering the area searched for DNA samples surrounded by an 8000 m buffer. The buffer width was calculated by multiplying by 4 the largest estimated σ in a preliminary analysis (Efford 2004).

Only samples found within the spatial bounds of the study areas (Fig. 1) for which coordinates, species, sex, and individual ID were available were considered a detection and assigned to the nearest detector. SCR datasets for each year and study area were built from the number of individual detections at each detector.

#### Model implementation and selection

We ran all models as multi-session spatial capture–recapture models using the oSCR package version 0.42.0 (Sutherland et al. 2019) in R version 3.6.0 (R core team 2019). The multi-session implementation allowed us to use data from different sessions, in this case years, in a single statistical model. This increases reliability and estimates effects on different parameters either jointly across sessions or independently (Sutherland et al. 2019).

We first constructed a simple multi-session model based on inherent study design specifications. We included session-specific density and detection probability to obtain year-specific estimates. To control for variable search effort along search transects, we also included a detector-specific covariate on p0, defined as the total length of registered GPS search tracks within each detector grid cell. To test for the effect of multiple covariates on red fox density, detection, and space use, we built and compared 16 extensions of the simple model, based on all possible combinations of the covariates of interest explained below.

To test for a relationship between red fox density and available forest habitat, as suggested by previously reported habitat preferences of the red fox (Cagnacci et al. 2004; Svendsen 2016; Van Etten et al. 2007), we considered an effect of forest cover as a spatial predictor of density (Molina et al 2017). Proportion of forest cover for each state-space grid cell was extracted in QGIS version 3.10 (QGIS Development Team 2019) based on maps at scales between 1:25 000 and 1:100 000 (Norwegian Mapping Authority 2020).

As density in SCR translates to the distribution of individual home ranges across the landscape, i.e., second-order habitat selection (Everatt et al. 2015), proportion of forest cover was defined as the average forest cover in a 1000 m radius around each raster cell.

To test for sexual dimorphism in red fox space use, we included sex as a predictor of σ and p0. Some studies report home-range size of the red fox to differ between sexes (Drygala and Zoller 2013), while other studies report no significant sex differences (Svendsen 2016; Walton et al. 2017). Because parameterization of the scale parameter affects baseline detection probability and vice versa (Efford and Mowat 2014), the sex effect was always tested simultaneously on σ and p0.

Furthermore, to account for additional sources of variation in detectability, we included road length and forest cover as predictors of p0. The inclusion of the road covariate was motivated by evidence suggesting that mesopredators often travel along roads in winter to conserve energy (Crête and Larivière 2003), while forest cover was included to test whether detectability was higher in open vs. covered areas. As an individual’s detection probability is tightly linked with space use within its home range, i.e., fourth-order habitat selection, all spatial covariates on p0 were extracted at the detector grid scale. Road length was thus defined as the total length of roads and forest cover as the average forest cover within each detector grid cell, based on maps at scales between 1:25 000 and 1:100 000 (Norwegian Mapping Authority 2020) Table 1.

**Table 1:** Summary of non-invasive genetic sampling data from red fox collected during field surveys in Lierne in central Norway (2016–2018) and in Skrim in southern Norway (2017–2018)

	Total no. of DNA samples	No. of red fox samples	No. of genotyped samples	No. of identified individuals	No. of identified females/males	Mean no. of samples per individual
Lierne 2016	160	76 (48%)	58 (36%)	26	19 / 7	2.23 (range: 1–8)
Lierne 2017	184	155 (84%)	95 (51%)	37	20 / 16	2.57 (range: 1–12)
Lierne 2018	158	152 (98%)	122 (77%)	27	12 / 15	4.52 (range: 1–17)
Skrim 2017	150	102 (68%)	43 (29%)	25	11 / 14	1.72 (range: 1–4)
Skrim 2018	133	121 (91%)	60 (45%)	25	12 / 13	2.40 (range: 1–6)

Fitted models were subjected to post-processing and model selection using functionality provided in the oSCR package. Model selection was performed using the Akaike Information Criterion (AIC; Burnham and Anderson 2002). For each study area, only the model with the lowest AIC value was retained for estimating density, population size, home range, and covariate effects (Table 2 and 3). Predicted values from the retained fitted models, including red fox density, realized density maps, and population size, were produced using functionality provided in the oSCR package (Sutherland et al. 2019). Additional detail is provided in Online Resource 2.

**Table 2:** Comparison of 16 red fox multi-session spatial capture–recapture models using Akaike Information Criterion (AIC), and the difference between AIC of each model and the model with the lowest AIC (ΔAIC)

*Density (D)*	*Detection (p0)*	*Sigma (σ)*	*No. of parameters*	*AIC*	*ΔAIC*
Session + forest	Session + effort + sex	Sex	14	1787.45	0.00
Session + forest	Session + effort + road + sex	Sex	15	1787.83	0.38
Session	Session + effort + road + sex	Sex	14	1789.30	1.85
Session + forest	Session + effort + sex + forest	Sex	15	1789.45	2.00
Session + forest	Session + effort + road + sex + forest	Sex	16	1789.74	2.29
Session	Session + effort + sex	Sex	13	1790.29	2.84
Session	Session + effort + road + sex + forest	Sex	15	1791.29	3.84
Session	Session + effort + sex + forest	Sex	14	1791.99	4.54
Session + forest	Session + effort	NA	12	1799.70	12.25
Session + forest	Session + effort + road	NA	13	1800.51	13.06
Session + forest	Session + effort + forest	NA	13	1801.69	14.24
Session + forest	Session + effort + road + forest	NA	14	1802.40	14.95
Session	Session + effort + road	NA	12	1803.94	16.49
Session	Session + effort	NA	11	1805.14	17.69
Session	Session + effort + road + forest	NA	13	1805.90	18.45
Session	Session + effort + forest	NA	12	1806.73	19.28

Covariates tested included a forest cover effect on density and detection; a road length effect on detection; and a sex effect on detection and space use, during a field survey in Lierne in central Norway (2016–2018). Covariates tested were extensions of a model that included study design-specific effects of year (session) on density and detection; and length of search transects (effort) on detection

**Table 3:** Comparison of 16 red fox multi-session spatial capture–recapture models in a candidate set using Akaike Information Criterion (AIC) and the difference between AIC of each model and the model with the lowest AIC (ΔAIC)

Density (D)	Detection (p0)	Sigma (σ)	No. of parameters	AIC	*ΔAIC*
Session	Session + effort + road + sex	Sex	11	761.27	0.00
Session	Session + effort + road + sex + forest	Sex	12	761.73	0.46
Session + forest	Session + effort + road + sex + forest	Sex	13	761.91	0.64
Session	Session + effort + sex	Sex	10	762.14	0.87
Session	Session + effort + road	NA	9	762.21	0.94
Session + forest	Session + effort + road + sex	Sex	12	762.48	1.22
Session	Session + effort + sex + forest	Sex	11	762.65	1.38
Session	Session + effort + road + forest	NA	10	762.71	1.45
Session + forest	Session + effort + sex + forest	Sex	12	762.80	1.53
Session + forest	Session + effort + road + forest	NA	11	762.92	1.66
Session	Session + effort	NA	8	763.07	1.81
Session + forest	Session + effort + sex	Sex	11	763.31	2.05
Session + forest	Session + effort + road	NA	10	763.34	2.07
Session	Session + effort + forest	NA	9	763.62	2.36
Session + forest	Session + effort + forest	NA	10	763.82	2.55
Session + forest	Session + effort	NA	9	764.17	2.90

The covariates tested included a forest cover effect on density and detection; road length effect on detection; and sex effect on detection and space use, during a field survey in Skrim in southern Norway (2017–2018). Covariates tested were extensions of a model that included study design-specific effects of year (session) on density and detection; and length of search transects (effort) on detection

## Results

### NGS samples

Out of 502 total samples collected in Lierne, 383 were confirmed as red fox, of which 275 samples were successfully assigned reliable genotypes and individual IDs (for a breakdown by sample type, see Table S1). Successfully genotyped samples originated from 98 different individuals. The mean number of samples per individual was 2.23 (range: 1–8) in 2016, 2.57 (range: 1–12) in 2017, and 4.57 (range: 1–17) in 2018 (Table 1). Out of 283 total samples collected in Skrim, 223 were confirmed as red fox, of which 103 were successfully assigned reliable genotypes and individual IDs. Successfully genotyped samples originated from 39 different individuals. The mean number of samples per individual was 1.72 (range: 1–4) in 2017 and 2.40 (range: 1–6) in 2018 (Table 1). A summary of the samples included in the SCR analysis is provided in Table S4.

### Model selection

The top model for Lierne included forest cover and year effects on density, year and search effort effects on baseline detection probability, and sex effects on both baseline detection probability and the scale parameter. For Skrim, the top model did not include an effect of forest cover on density. Baseline detection probability was influenced by year, search effort, and road length and fox sex influenced both baseline detection probability and the scale parameter.

### Estimated population size and density

**Fig. 2:**
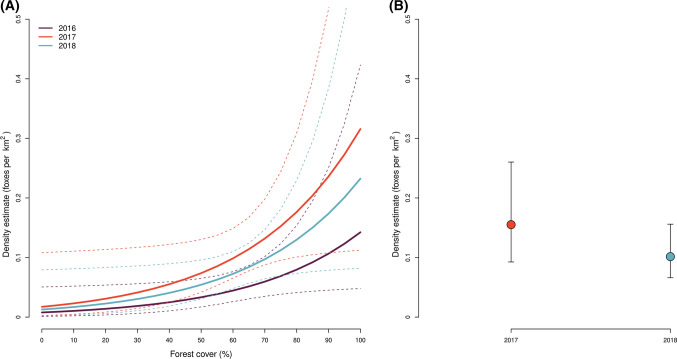
Estimated red fox density from the best-supported spatial capture–recapture models based on non-invasive DNA samples from Lierne, central Norway (2016–2018) and Skrim, southern Norway (2017–2018). The best-supported model for Lierne included effects of year and forest cover on red fox density (**A**) and only differences between years in Skrim (**B**). Plots present the mean predicted densities (colored lines in **A** and point in **B**) and associated 95% confidence intervals (dashed lines in **A** and whiskers in **B**)

Estimated average red fox densities across the study area in Lierne (Fig. 1A) were 0.04 foxes per km^2^ in 2016, 0.10 in 2017, and 0.06 in 2018. Furthermore, density was predicted to increase with forest cover (β_forest_ = 2.91 [95%CI 0.14–5.67]; Fig. 2A; Table S5). Mean-estimated population size within the original 225 km^2^ study area in Lierne (Fig. 1A) was 9 foxes in 2016, 22 in 2017, and 14 in 2018.

**Fig. 3:**
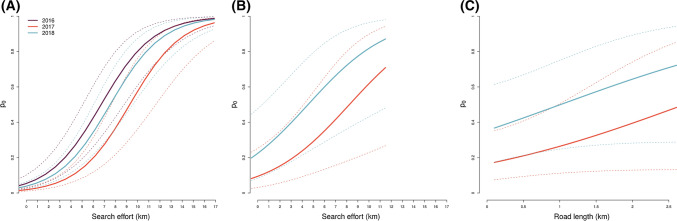
Estimated red fox baseline detection probability (p_0_) from the best-supported spatial capture–recapture models based on non-invasive DNA samples from Lierne, central Norway (2016–2018) and Skrim, southern Norway (2017–2018). The best-supported model for Lierne included effects of year and search effort on red fox baseline detection probability (**A**), while the best model for Skrim included effects of year, search effort (**B**), and road length (**C**). Both models also included a sex effect; presented are predicted p0 values for males. Coloured lines represent the mean predicted values and dashed lines represent the associated 95% confidence intervals

**Fig. 4:**
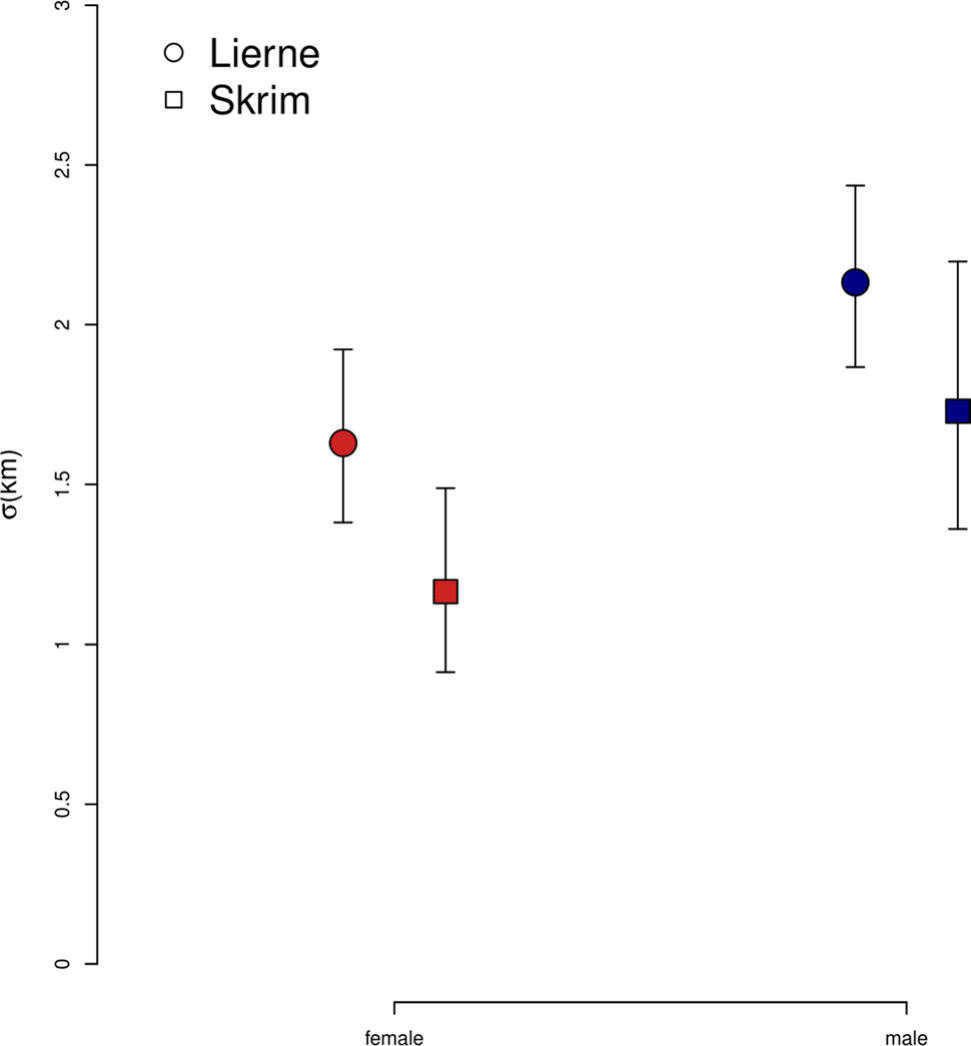
Estimated red fox scale parameter (σ) from the best-supported spatial capture–recapture models based on non-invasive DNA samples from Lierne (coloured circles) and Skrim (coloured squares), Norway. The best-supported model for both Lierne and Skrim included a difference between sexes. Dots represent the mean values and whiskers represent the associated 95% confidence intervals

**Fig. 5:**
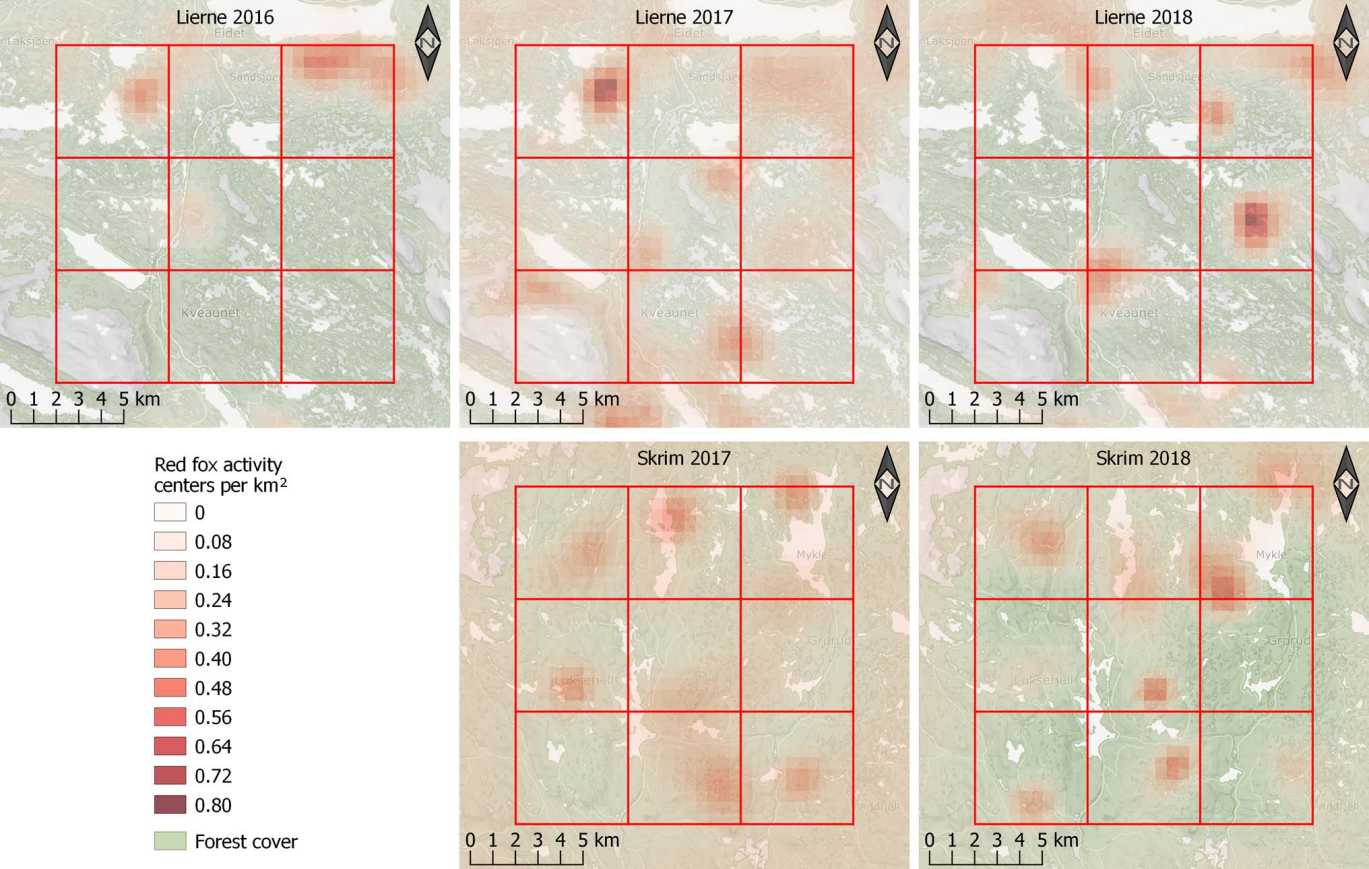
Annual realized red fox density maps derived from the best-supported spatial capture–recapture models based on non-invasive DNA samples from Lierne, central Norway in 2016, 2017, and 2018, and from Skrim, southern Norway in 2017 and 2018

Estimated average red fox densities across the study area in Skrim (Fig. 1B) were 0.16 and 0.09 foxes per km^2^ in 2017 and 2018, respectively (Fig. 2B). These estimates corresponded to population sizes of 36 foxes and 20 foxes within the 225 km^2^ study area in 2017 and 2018, respectively (Fig. 1B). Mean-estimated densities for each study area per year are shown in Figs. 3, 4, and 5.

### Detection and space use

In Lierne, baseline detection probability increased with search effort (β_search_ = 0.76 [0.60–0.92]; Fig. 3A) and was higher for males than for females, albeit not significantly (β_sexMale_ = 0.32 [ – 0.23–0.88]; Table S5). In Skrim, baseline detection probability increased with both search effort (β_search_ = 0.33 [0.12–0.54]; Fig. 3B) and road length (β_road_ = 0.2 [0.03–0.43]; Fig. 3C). In addition, baseline detection probability was non-significantly higher for females than for males in Skrim (β_sexMale_ =  – 0.76 [ – 1.71–0.19]; Table S6).

In Lierne, σ estimates were 1.63 [1.38 -1.92] km for females and 2.13 [1.87–2.44] km for males (Fig. 4), which corresponded to home-range sizes of 50 [36–69] and 86 [66–112] km^2^ for females and males, respectively. In Skrim, σ estimates were 1.17 [0.91–1.49] km for females and 1.73 [1.36–2.19] km for males (Fig. 4), corresponding to home-range sizes of 26 [16–42] km^2^ and 56 [35–91] km^2^, respectively.

## Discussion

Ecologists and wildlife managers have a keen interest in quantifying wildlife abundance across landscapes and identifying the drivers of spatial variation therein (Bischof et al. 2020). The ability to accomplish these goals has gained spatial capture–recapture analysis substantial popularity over the past decade (Royle et al. 2018). SCR has proven a particularly powerful tool when used in concert with non-invasive methods as this has allowed population estimation at a scale and with a level of detail that had until recently been unattainable (Bischof et al. 2020).

Despite being the most widespread carnivore species globally, there is a paucity of detailed information about red fox population densities and their determinants (Wegge et al. 2019). Using non-invasive genetic sampling and SCR analysis, we mapped the density of red foxes in two boreal forest landscapes in Norway over 3 years. Our study revealed that a combination of spatial and individual factors influences density, space use, and detection probability.

We estimated higher red fox densities in the southern study area (Skrim) compared to the forest in central Norway (Lierne). Higher altitude, higher latitude, a more continental climate, and lower winter temperatures make Lierne less productive than Skrim (Moen 1998). The difference in estimated red fox densities between the study areas may partially be attributed to difference in vegetation and climate (Walton et al. 2017), and winter severity as limiting factors on density (Bartoń and Zalewski 2007). Human land use and anthropogenic subsidies have also been suggested to be important drivers of red fox density (Gomo et al. 2017; Rød-Eriksen et al. 2020). Forest landscapes with high human settlement density are associated with higher red fox abundances, potentially driven by increased food availability of anthropogenic origin, and thus increased scavenging opportunities (Jahren et al. 2020; Rød-Eriksen et al. 2020). Due to a larger human population as well as more clusters of cabins in adjacent areas in Skrim compared to Lierne, differences in density estimates may partially reflect differences in human influence.

The two study areas also differed in terms of variables that predicted red fox density. We detected a significant positive effect of forest cover on red fox density in Lierne but not in Skrim. Boreal forests are important habitats for several prey species of red fox, including voles, shrews, and forest birds (Needham et al. 2014). Forests likely also provide important refuges in winter in contrast to more exposed alpine areas. The lack of an effect of forest cover in Skrim may reflect a paucity of evidence, perhaps because variation in forest cover was very low, with less open unforested areas like bogs and impediment (Fig. S4). We note that several candidate models were close in support based on AIC in both study areas (Tables 2 and 3). However, additional covariate effects included in these models did not explain enough variation to justify their inclusion and were therefore not interpreted as having an ecological effect (Arnold 2010).

SCR models allowed us to derive sex-specific home-range sizes. The approach implemented here relies on the assumption of a normally distributed circular home range, and we note that SCR-derived home-range estimates have been shown to be sensitive to misspecification of the detection function (Royle et al. 2014). Nevertheless, our home-range size estimates for both study areas were comparable to estimates from two recent GPS telemetry studies of red fox in similar habitat in Scandinavia. Svendsen (2016) reported mean red fox home-range size of 61 km^2^ [95% CI 25–105] for the region of Østerdalen, Innlandet, and Walton et al. (2017) reported mean home-range sizes of 52 km^2^ [95% CI 32–72] for the regions of Kolmården, Grimsö, and Hedemora in Sweden, and Hedmark in Norway. However, the variation in reported individual home-range estimates was significant in both studies. Walton et al. also reported home ranges up to four times larger in less-productive and high elevation landscapes compared to more productive and low elevation landscapes (2017). We found a similar pattern with smaller home ranges in the more productive lower elevation southern boreal forest (26 [16–42] km^2^ for females and 56 [35–91] km^2^ for males in Skrim), compared to Lierne’s less-productive higher elevation northern boreal forest (45 [34–60] km^2^ for females and 88 [69–113] km^2^ for males). In contrast to studies by Svendsen (2016) and Walton et al. (2017), which reported no differences in home range between males and females, our study found home-range estimates of males to be approximately twice the size of females in both study areas. This may reflect variation in space use related to breeding status of females, as reproductive females have been reported to have smaller home ranges (Henry et al. 2005). Some females may have started retreating to natal dens towards the end of the sampling period (Walton and Mattisson 2021), which would affect their home-range sizes. Furthermore, DNA sampling was partly done during the mating period (January–March), when male foxes likely roam around to cover several female home ranges (Cavallini 1996), which could contribute to the observed difference between males and females*.*

One important advantage of SCR is that it accounts for imperfect and variable detection of individuals. Though many count-based wildlife surveys assume complete detection of all individuals in a population, this assumption is almost always violated (Kellner and Swihart 2014). When not accounted for, imperfect detection can lead to erroneous inferences about density and its drivers (Gu and Swihart 2004). In addition, in most monitoring set-ups, detection probability differs amongst individuals in the population as a result of different exposure to detectors in relation to individual home-range locations (Efford and Mowat 2014). SCR models use this inherent heterogeneity in detectability to estimate individual activity centres and space-use patterns (Royle et al. 2013). In our study, variation in detection probability was also influenced by spatial predictors, including a positive effect of search effort effect in both areas, and a positive trend of an effect of road length in Skrim only (Table S6). Lack of an effect of roads in Lierne may be due to insufficient evidence, as roads were fewer and covered less of the study area (Fig. S3). Detection probability also differed between years. Given that the detection of individual animals depended on the genetic analysis of NGS samples, this may reflect variation in genotyping success rates between years (Table 1).

Forty-eight percent of all samples collected in our study contained DNA of sufficient quality for individual identification. The proportion of successfully genotyped samples was noticeably higher in Lierne compared to Skrim, and fecal samples had the highest genotyping success rates in both areas (Table 1 and S1, Fig. S1). Many factors, including sample age, temperature, moisture, and UV radiation, contribute to degradation and preservation of DNA (Hausknecht et al. 2007; Panasci et al. 2011; Piggott 2005; Woodruff et al. 2015). The reported differences in genotyping success rates may thus reflect differences in environmental conditions. Considering that the samples collected were of varying type and quality, the genotyping success rates reported here validate the NGS methods as viable for identifying individual foxes. However, we also want to highlight the possibilities of implementing other types of data into the SCR framework for future studies. Multiple data sources, such as recoveries of dead animals, can also be integrated in the SCR framework to increase the precision of estimates (Dupont et al. 2021). Several methods were recently proposed for incorporating detections of unidentified individuals, leading to more precise estimation (Jiménez et al. 2019, 2021; Tourani et al. 2020).

A similar study by Wegge et al. (2019) produced red fox density estimates using SCR, but argued that a main shortcoming of their study was a smaller sampled area (50 km^2^). Because the scale parameter relates to home-range size, parameter estimates including density are more likely to be biased when the sampled area is small relative to the range of individual movements in the study population, particularly if sample size is low (Sollmann et al. 2012; Sun et al. 2014). We thus want to reemphasize the importance of considering spatial detector configuration relative to the known space use and home-range size of the study species.

Though we obtained density estimates that varied between years, the closed population SCR approach applied here is less suitable for studying the drivers of inter-annual differences in red fox density. Recent developments of SCR methods include open population analyses that allow for studying red fox population dynamics over time, including estimating mortality and recruitment rates, as well as immigration and emigration (Morin et al. 2016). This would also make better use of the available data, as information on individual states is propagated between years (Ergon and Gardner 2014; Gardner et al. 2018; Milleret et al. 2020).

The combination of SCR and NGS methods provides a solid framework not only for estimating red fox density, but also to identify drivers thereof (e.g., productivity, snow depth, forest cover, and influence of human activity). If applied at larger scales in different habitats, e.g., mosaics of forest and farmland or arctic and alpine areas, this approach has the potential to provide new insight into the relative importance of various drivers of red fox population dynamics.

## Supplementary Information

Below is the link to the electronic supplementary material.

Supplementary file1 (PDF 1294 KB)
