# Supplementary material for: Estimating red fox density using non-invasive genetic sampling and spatial capture–recapture modelling
Source: Oecologia. 2021 Dec 2;198(1):139–51. doi: 10.1007/s00442-021-05087-3 (PMC8803778; doi:10.1007/s00442-021-05087-3)
Supplement: Supplementary file 1 — Supplementary file1 (PDF 1294 KB) [file 442_2021_5087_MOESM1_ESM.pdf]

Online Resource 1 - Appendices

Estimating red fox density using non-invasive genetic sampling and spatial capture-recapture modelling

Lars K. Lindsø,[abc] Pierre Dupont,[b] Lars Rød-Eriksen,[a] Ida Pernille Øystese Andersskog,[a] Kristine Roaldsnes Ulvund,[a] Øystein Flagstad,[a] Richard Bischof,[b] Nina E. Eide[a]

[a]Norwegian Institute for Nature Research, Department of Terrestrial Ecology, Høgskoleringen 9, 7034 Trondheim, Norway
[b]Norwegian University of Life Sciences, Faculty of Environmental Sciences and Natural Resource Management, Universitetstunet 3, 1430 Ås, Norway
[c]Centre for Ecological and Evolutionary Synthesis (CEES), the Department of Biosciences, University of Oslo, Blindernveien 31, 0371 Oslo, Norway

Corresponding author: Lars K. Lindsø lars.lindso@ibv.uio.no

Table S1: Number of genetic samples found by and the number (proportion) of red fox samples that were successfully genotyped during genetic sampling study in Lierne, central Norway in 2016, 2017, and 2018 and in Skrim, southern Norway in 2017 and 2018. * = No urine preservatives were used in the pilot study (Lierne 2016).

	**Scats**		**Urine**		**Hair**	
	Found	Genotyped	Found	Genotyped	Found	Genotyped
Lierne 2016	60	56 (93 %)	100	*2 (2 %)	0	0 (N/A)
Lierne 2017	69	53 (77 %)	98	40 (41 %)	17	2 (12 %)
Lierne 2018	78	74 (95 %)	76	47 (62 %)	4	1 (25 %)
Skrim 2017	69	23 (33 %)	81	20 (25 %)	0	0 (N/A)
Skrim 2018	38	37 (97 %)	93	23 (25 %)	2	0 (0 %)
Total	314	243 (77 %)	448	130 (29 %)	23	3 (13 %)

Table S2: Primer set concentrations in micromolar (µM) for the species specificity test and individual identification of confirmed red fox samples (MIX A + MIX B).

**Species determination**		**Multiplex MIX A**		**Multiplex MIX B**	
**Locus**	**µM**	**Locus**	**µM**	**Locus**	**µM**
gulo	0.12	*AHT133*	0.24	*AHTh171*	0.16
pex	0.12	*C08.618*	0.12	*CPH11*	0.12
vul	0.12	*CPH2*	0.16	*CPH18*	0.16
H3R	0.36	*FH2001*	0.24	*CPH7*	0.04
		FH2054	0.10	*CXX-468*	0.12
		FH2328	0.24	*FH2848*	0.16
				K9-AMELO	0.08
				REN54P11	0.24

Table S3: Microsatellite markers used in the analysis of genetic samples in Lierne, central Norway in 2016, 2017, and 2018 and in Skrim, southern Norway in 2017 and 2018, with respective multiplex panels, dye colors, allele number and size range (bp = base pairs). * = sex marker

Marker	***Mplex***	***5' label***	***Allele number***	***Allele range (bp)***
AHT133	MIX A	Pet	9	85-111
C08.618	MIX A	Pet	7	190-202
CPH2	MIX A	Ned	8	93-111
FH2001	MIX A	6-Fam	4	132-150
FH2054	MIX A	Ned	7	142-198
FH2328	MIX A	Vic	9	131-167
FH2457	MIX A	Vic	11	280-323
AHTh171	MIX B	Pet	5	155-167
CPH11	MIX B	6-Fam	3	114-118
CPH18	MIX B	6-Fam	8	168-192
CPH7	MIX B	Ned	4	154-176
CXX-468	MIX B	Vic	7	82-94
FH2848	MIX B	Vic	6	221-235
*K9-Amelo**	MIX B	Ned		202, 216
REN54P11	MIX B	Pet	9	189-219

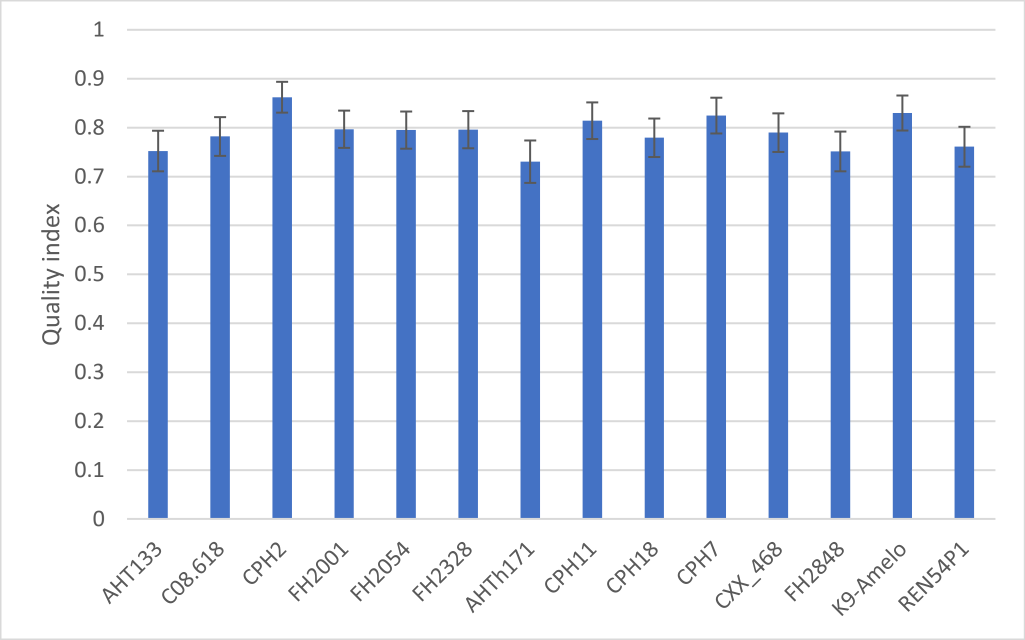

Figure S1: Mean quality index (QI) per locus in Lierne. Error bars denote the 95 % confidence interval. Data was obtained through non-invasive genetic sampling of red fox scats, urine and hair from a field survey in Lierne, central Norway in 2016, 2017, and 2018.

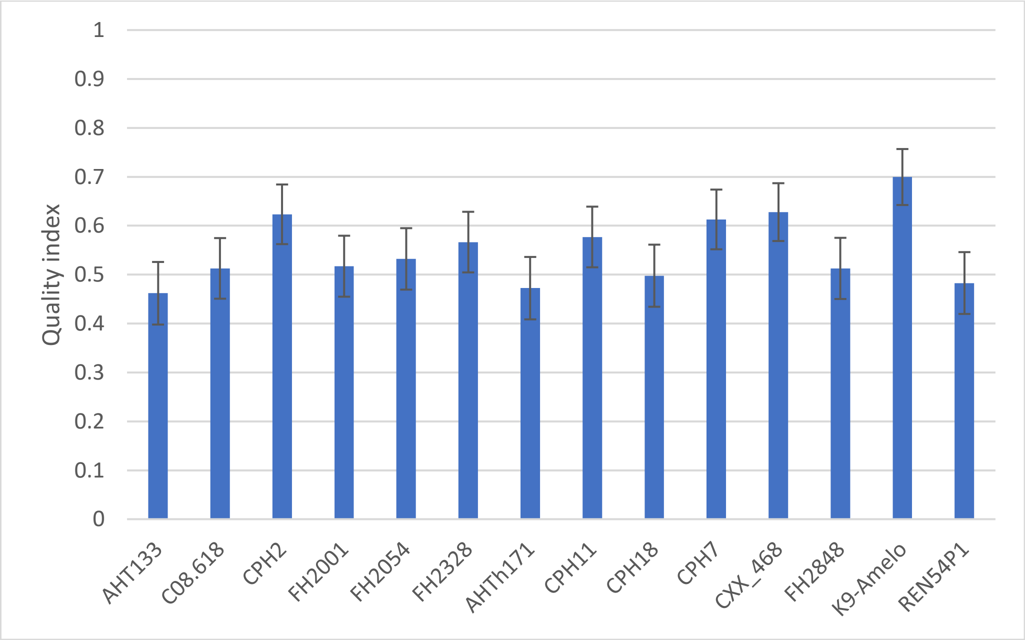

Figure S2: Mean quality index (QI) per locus in Skrim. Error bars denote the 95 % confidence interval. Data were obtained through non-invasive genetic sampling of red fox scats, urine, and hair during a field survey in Skrim, southern Norway in 2017 and 2018.

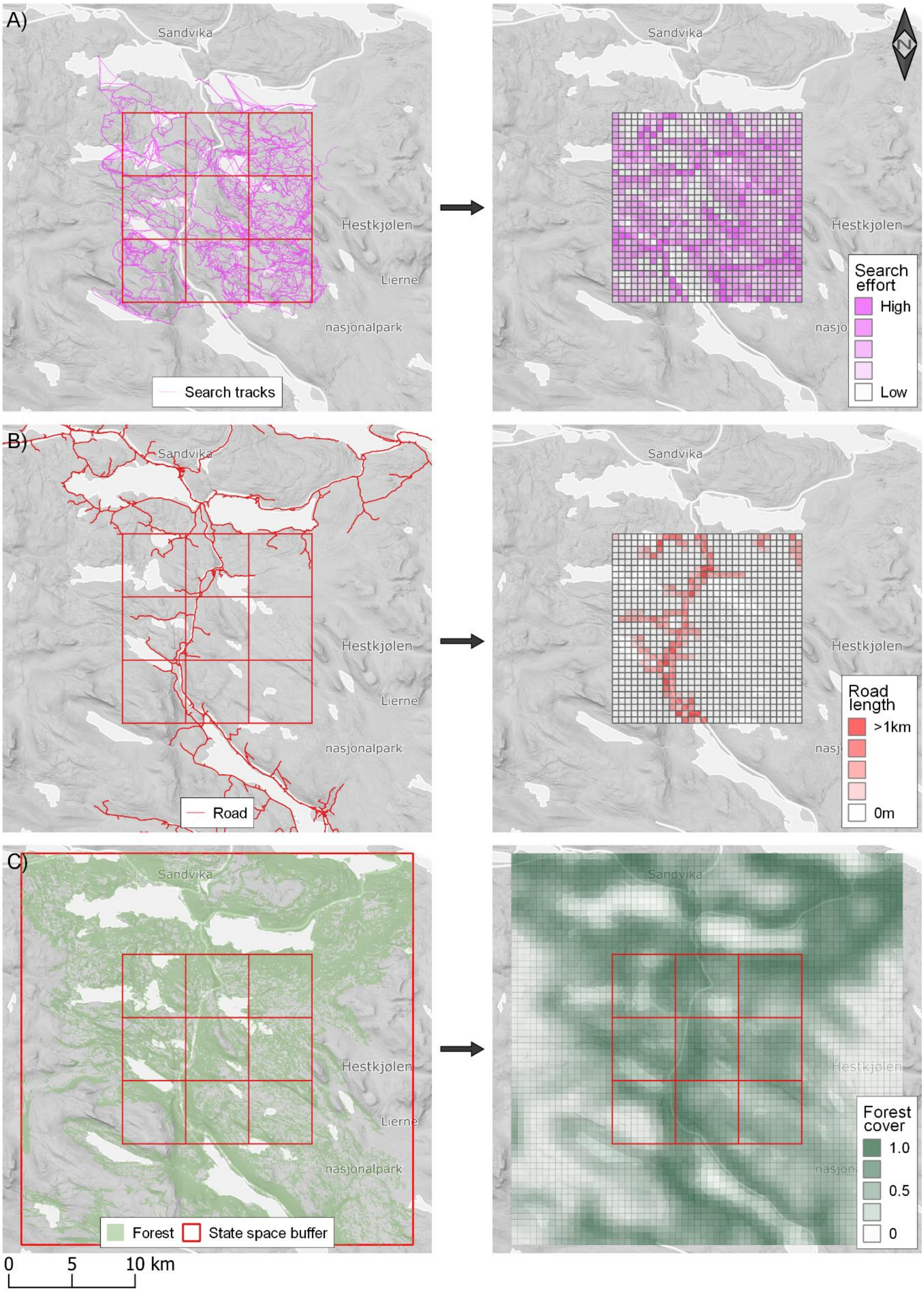

Figure S3: Maps of spatial covariates used in the red fox spatial-capture recapture analysis of non-invasive genetic sampling data collected in Lierne, central Norway in 2016, 2017, and 2018:

A) Search effort per detector from length of GPS search transects.

B) Road length per detector from N50 road data (Norwegian Mapping Authority 2020).

C) Forest cover per state-space cell from N50 forest data, extracted in a 1000 m radius around each state-space cell (Norwegian Mapping Authority 2020).

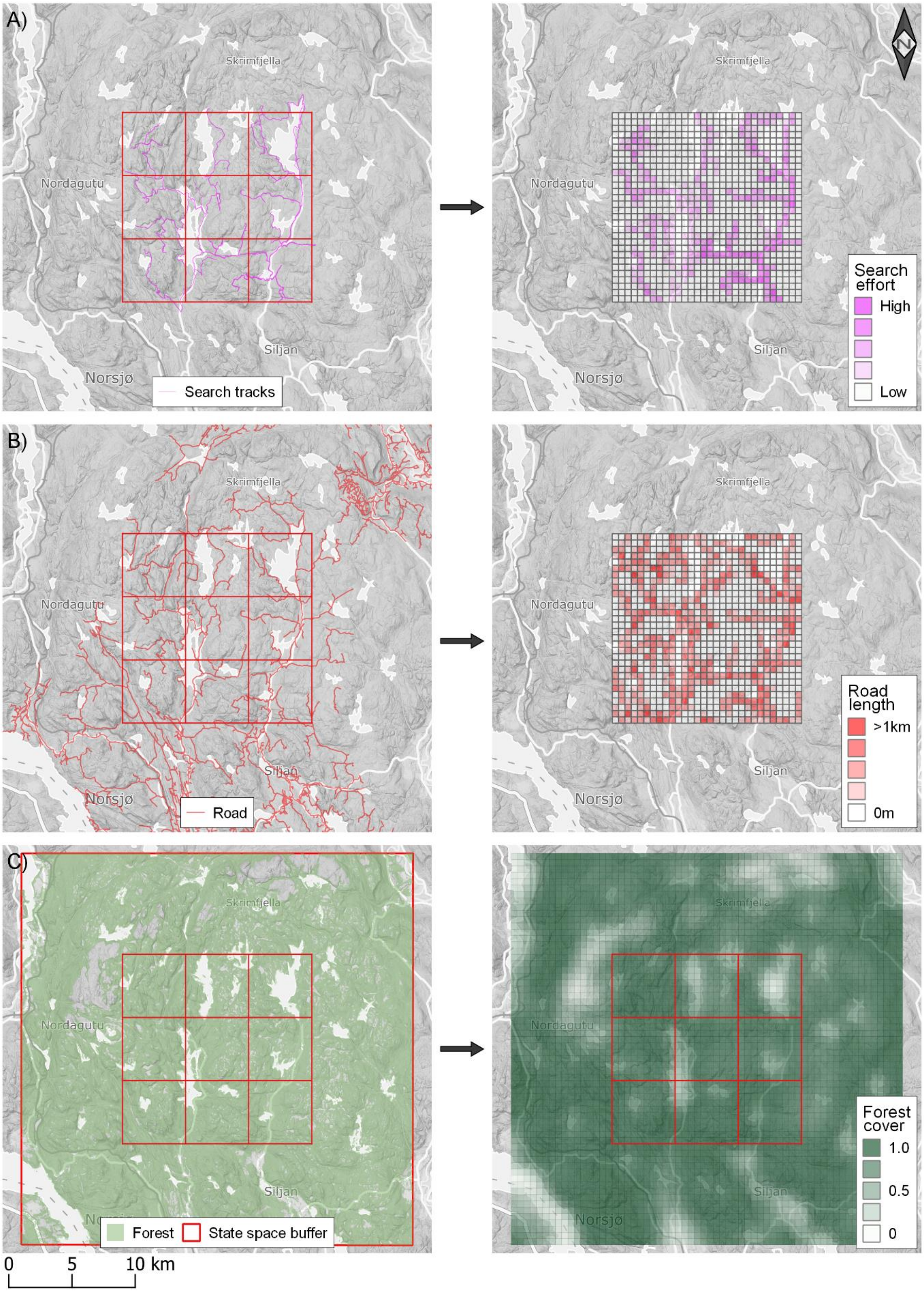

Figure S4: Maps of spatial covariates used in the red fox spatial-capture recapture analysis of non-invasive genetic sampling data collected in Skrim, southern Norway in 2017 and 2018:

A) Search effort per detector from length of GPS search transects.

B) Road length per detector from N50 road data (Norwegian Mapping Authority 2020).

C) Forest cover per state-space cell from N50 forest data, extracted in a 1000 m radius around each state-space cell (Norwegian Mapping Authority 2020).

Table S4: Summary of capture data used in red fox multi-session spatial capture-recapture models. The capture data was obtained through non-invasive genetic sampling of red fox scats, urine, and hair during a field survey in Lierne, central Norway in 2016, 2017, and 2018 and in Skrim, southern Norway in 2017 and 2018.

	**No. of individuals**	**No. of females/males**	**Average no. of spatial detections**
Lierne 2016	15	9/6	2.33 (range: 1-7)
Lierne 2017	29	16/13	2.48 (range: 1-12)
Lierne 2018	26	11/15	3.58 (range: 1-17)
Skrim 2017	24	11/13	1.63 (range: 1-4)
Skirm 2018	24	12/12	2.08 (range: 1-5)

Table S5: Coefficient table for model parameters in the top red fox spatial-capture recapture model during a field survey in Lierne, central Norway in 2016, 2017, and 2018; baseline detection probability (p0), scale parameter (σ) and density (D).

	**Estimate**	**SE**	**z**	**P(>|z|)**
p0(Intercept)	-2.513	0.342	-7.339	0.000
$p0\beta_{year2017}$	-1.098	0.353	-3.113	0.002
$p0\beta_{year2018}$	-0.348	0.351	-0.990	0.322
$p0\beta_{search}$	0.762	0.082	9.274	0.000
$p0\beta_{sexMale}$	0.324	0.283	1.144	0.253
σ(Intercept)	7.396	0.084	87.557	0.000
$\sigma\beta_{sexMale}$	0.269	0.108	2.486	0.013
D(Intercept)	-6.243	0.960	-6.505	0.000
$D\beta_{forest}$	2.908	1.411	2.061	0.039
$D\beta_{year2017}$	0.796	0.331	2.401	0.016
$D\beta_{year2018}$	0.490	0.333	1.472	0.141

Table S6: Coefficient table for model parameters in the top red fox spatial-capture recapture model during a field survey in Skrim, southern Norway in 2017 and 2018; baseline detection probability (p0), scale parameter (σ) and density (D).

	**Estimate**	**SE**	**z**	**P(>|z|)**
p0(Intercept)	-2.789	0.430	-6.487	0.000
$p0\beta_{sexMale}$	-0.758	0.484	-1.568	0.117
$p0\beta_{year2018}$	1.020	0.370	2.757	0.006
$p0\beta_{search}$	0.331	0.106	3.112	0.002
$p0\beta_{road}$	0.200	0.117	1.714	0.087
σ(Intercept)	7.061	0.124	56.721	0.000
$\sigma\beta_{sexMale}$	0.394	0.174	2.272	0.023
D(Intercept)	-3.250	0.264	-12.316	0.000
$D\beta_{year2018}$	-0.425	0.339	-1.253	0.210
